# Patient perceptions of symptoms and concerns during cancer chemotherapy: ‘affects my family’ is the most important

**DOI:** 10.1007/s10147-017-1117-y

**Published:** 2017-04-06

**Authors:** H. Sasaki, K. Tamura, Y. Naito, K. Ogata, A. Mogi, T. Tanaka, Y. Ikari, M. Masaki, Y. Nakashima, Y. Takamatsu

**Affiliations:** 1 Division of Medical Oncology, Hematology and Infectious Diseases, Department of Medicine, Fukuoka University Hospital, 7-45-1 Nanakuma, Jonan-ku, Fukuoka, 814-0180 Japan; 2 General Medical Research Center School of Medicine, Fukuoka University, Fukuoka, Japan; 3 Department of Pharmacology, Fukuoka University Hospital, Fukuoka, Japan

**Keywords:** Chemotherapy, Side effects, Pain, Patient perceptions

## Abstract

**Background:**

Cancer chemotherapy is associated with a variety of side effects/adverse events. It is very important that patients adhere to the planned chemotherapy regimen, which necessitates a minimum of side effects and that these side effects be kept under control. We have investigated patients’ concerns and symptoms during chemotherapy with the aim to seek solutions that will improve patients’ quality of life during chemotherapy.

**Methods:**

Forty-nine patients with malignant diseases on parenteral antineoplastic agents were sequentially enrolled in this study. These patients completed a questionnaire consisting of 42 items related to non-physical concerns and 52 items of physical symptoms related to chemotherapy. Each patient was also asked to select the three items among these 94 items which affected him/her the most.

**Results:**

The median age of the cancer patients was 62 years and the male-to-female ratio was 18:31. Among the non-physical concerns, the most frequently chosen concern was ‘affects my family or partner,’ followed by anxiety related to treatment. Regarding the physical symptoms, the most frequent complaints were fatigue, alopecia and constipation, while the most troublesome symptoms were nausea, poor taste and paresthesia. Overall, the most frequently expressed concerns were ‘affects my family or partner’ and anxiety related to treatment. Male patients suffered most from fever, fatigue and nausea, and female patients complained more of poor taste and gastrointestinal problems.

**Conclusion:**

Patient perceptions of adverse events associated with cancer chemotherapy apparently have changed from physical symptoms to non-physical concerns. In our patient cohort ‘affects my family or partner’ was the most important concern. One important point to note is that female patients often complained of poor taste because this meant they were unable to cook well.

**Electronic supplementary material:**

The online version of this article (doi:10.1007/s10147-017-1117-y) contains supplementary material, which is available to authorized users.

## Introduction

Cancer has been a leading cause of death in Japan since 1981, and one out of two Japanese people develops cancer during their life time [1]. Early detection and treatment are essential to cure this devastating disease. However, with a few exceptions, such as lymphoma, leukemia and germ cell tumors, advanced and relapsed cancers are rarely curable by any treatment modalities. Even patients who are thought be cured have had to go through painful laboratory examinations and treatments consisting of surgery and/or radiation therapy and/or chemotherapy. Surgery and irradiation are local treatments that are completed within a couple of weeks, and usually only the local adverse sequelae are significant. In contrast, chemotherapy is scheduled over months or even years in some diseases. It is a systemic therapy often associated with a variety of side effects and can occasionally be life-threatening. The emergence of adverse side effects can negatively affect a patient in a variety of ways. Consequently, there is a need to control such side effects since interruption of the chemotherapeutic regimen is detrimental to both a high cure rate or prolongation of survival with a good quality of life (QoL).

In 1983, an Australian research roup reported patients’ perceptions of the side effects of cancer chemotherapy. Emesis, nausea and loss of hair were identified as major physical symptoms, and anxiety when coming to treatment, treatment time and fear of needles were major non-physical concerns [2]. Ten years later, following the introduction of a new group of antiemetics, the 5-hydroxytryptamine 3 (5HT3) receptor antagonists into clinical use, the same research group conducted a repeat study using the same measures. This time, nausea overtook emesis, which ranked fifth in the repeat study, and there was a clear shift in patient concerns from those about physical symptoms to psychosocial issues [3]. In 2002, a French group carried out a similar survey and found that chemotherapy-induced nausea and vomiting (CINV) was no longer within the top ten of patient concerns and that ‘affects my family or partner’ ranked first [4].

In Japan, aprepitant, a neurokinin-1 (NK-1) receptor antagonist, and palonosetron, a second-generation 5HT3 receptor antagonist, were approved for in 2009 and 2010, respectively, and their use has gradually spread throughout Japan. By 2011 these new antiemetics were routinely used in medical practice. It is therefore timely to investigate patients’ perceptions of the side effects and related issues of cancer chemotherapy.

## Methods

### Patients and methods

We sequentially selected patients with pathologically documented malignant disorders who were being treated with parenteral antineoplastic agents in our department. All solid tumor patients had advanced cancer, and all hematological malignancy patients were at advanced stages. Patients who had received cytotoxic chemotherapy in the 4-week period immediately prior to their interview were included in the study. The study was approved by the Fukuoka University Hospital Review Board. A cancer research nurse explained the aim and design of the study to the patients. Those who consented to participate were asked to complete a questionnaire consisting of 94 items on signs and symptoms potentially related to chemotherapy. Upon completion by the patient, the nurse checked whether all answers were appropriately filled in and asked the patient to correct any entry which was not completed or the answer unclear. This questionnaire consisted of two categories of questions, group A and group B; for each item of group A the patient was asked to choose between yes or no as answer, and for the items in group B, the patient was asked to choose one of five options, from the worst symptom (grade 4) to no complaint (grade 0). The patient was also requested to choose the three items that influenced/affected him/her most, i.e., the most painful or most troubling events from group A, group B, and the entire 94 items. Most patients were able to choose three items which were most troublesome or painful. Patients who had difficulty in choosing three items were offered help from the nurse in the form of explanations. These nurses were members of the nursing staff who had no association with the patients in routine clinical practice, and they intervened as little as possible while the patient was filling in the questionnaire.

### Creating a questionnaire

To be able to compare the perceptions of our Japanese patients with those of patients in Western countries, we used the questions covered by Carelle et al.’s study [4], which used a questionnaire with items on 45 physical symptoms and 27 non-physical concerns. Since there no studies on patient perceptions of symptoms and concerns who received chemotherapy in Japan have been conducted, we had those items translated into Japanese. Some items had to be changed and/or added to in order to facilitate ease of answering for Japanese people. We changed “physical concerns” to descriptions easy for Japanese patients to understand; for example, for “pain” we added items to each specific site. We then rearranged the items in anatomical order, again to make it easier for the patient to understand the question. For “non-physical concerns”, we changed items to correlate better with present-day life in Japan. Specifically, items of aging, social and economic problems, and human relations were modified and/or added. Finally, we added 11 and 26 items for “physical concerns” and “non-physical concerns”. Thus, while several items have been added to those in questionnaire used in Carelle et al.’s study [4], all of their items were included, making it possible to compare our results with those of previous studies in general and Carelle et al.’s study [4] in particular. The questionnaire which we created consisted of a group of questions pertaining to non-physical concerns (group A) and a group of questions pertaining to physical concerns (group B) (Tables 1, 2). Items in both group A and group B related to potential adverse events of chemotherapy. The Japanese version of the questionnaire is given in the Electronic Supplementary Material.

**Table 1:** Group A items in the questionnaire—non-physical concerns

No. of item	Concerns	No. of item	Concerns
1	Forget things	22	When do I have to go to a hospice care facility?
2	Feeling low	23	Cannot get clothes that fit
3	Feeling sick	24	Affects my work/home duties
4	Easily excited	25	Affects my family or partner
5	Cannot move forward	26	Can I return to my normal social life?
6	Losing the will	27	Affects social activity
7	Whimper/crying more often	28	Length of time treatment takes at clinic
8	Irritable	29	Presence of family members I have to take care of
9	Cannot concentrate	30	Cannot eat raw foods
10	Feeling fearful	31	Can I get married?
11	Fear of death	32	Effects delivery or infants or raising my children
12	Hard to understand what is going on	33	Infertility
13	Feeling anxious about treatment	34	Loss of sexual ability
14	Feeling anxious about being unable to recover from the previous therapy	35	Travel time to come to the hospital
15	Feeling anxious about my life	36	Medical costs
16	No adviser	37	Relationships with medical staff
17	No supporter	38	Relationships with other patients
18	I do not want others to know about my disease	39	Problem with the hospital
19	Changing the primary physician	40	Contact methods for medical staff
20	Can I trust information on the internet?	41	Can I call medical staff when I am sick?
21	Hesitant to ask my primary physician to get a second opinion	42	Others ()

Please select the three most troubling items from items 1 to 42: (), (), ()

**Table 2:** Group B items in the questionnaire—physical symptoms

No. of item	Concerns	No. of item	Concerns
43	Nausea	69	Pain during urination
44	Emesis	70	Difficulty in urination
45	Constipation	71	Nasal bleeding
46	Diarrhea	72	Headache
47	Change in the way things taste	73	Dizziness
48	Taste nothing	74	Giddiness on standing up
49	Poor appetite	75	Ringing in ears
50	Sore throat	76	Difficulty in hearing
51	Abdominal pain	77	Bruise easily
52	Abdominal distension	78	Urticaria/wheals
53	Dry mouth	79	Change in skin color
54	Change in how things smell	80	Change in nails (color, pain, breaks)
55	Stomatitis/sore mouth	81	Hot flush
56	Increase appetite	82	Acne (pimples) or eczema
57	Cheilosis	83	Dry skin
58	Difficulty in swallowing	84	Itching
59	Numbness (Which part of body?)	85	Dermatitis sensitive to sun
60	Pain in fingers and/or toes	86	Fever
61	Dulling of senses	87	Fatigue
62	Tingling sensation (Which part of body?)	88	Alopecia/loss of hair
63	Pain at injection site	89	Weight loss
64	Poor veins	90	Weight gain
65	Anthralgia	91	Edema
66	Myalgia	92	Palpitation
67	Colored urine	93	Shortness of breath
68	Amount of urine	94	Insomnia

Please select the three most troubling items from 43 to 94: (), (), ()

## Results

A total of 49 cancer patients receiving chemotherapy as an outpatient or an inpatient at Fukuoka University Hospital participated in this study during a period of 6 months from September 2011 to February 2012. The background characteristics of these patients are shown in Table 3. The median age was 62 (range 32–84) years, and the male-to–female ratio was 18:31. There were 21 patients with hematological malignancies and 28 with solid tumors. The anticancer agents used in 36 (74%) patients were categorized as highly or moderately emetogenic according to the CINV guidelines in Japan [5].

**Table 3:** Patient demographics and clinical characteristics

Characteristics	Value
No. of patients	49
Age (years)	62 [32–84]
Gender	
Male	18
Female	31
Disease types	
Hematological malignancy	21
Malignant lymphoma	10
Multiple Myeloma	4
Acute leukemia	5
Others	2
Solid tumor	28
Breast	9
Colorectal	5
Esophagus	3
Stomach	3
Lung	2
Others	6
Emetic risk	
High	16
Moderate	20
Low	7
Minimal	6

Values in table are presented as the median with the range in square brackets or as a number

Patients with lymphoma, leukemia and breast cancer mainly received an anthracycline-based regimen while those with colorectal or lung cancer were treated with oxaliplatin or carboplatin combination chemotherapy.

A total of 91 symptoms were noted to be adverse events by the patients. Frequent painful non-physical concerns were ‘affects my family or partner’ followed in order by anxiety about difficulty in recovering from the previous chemotherapy, ‘affects my work or housework’, sick feeling, dissatisfaction about a change of primary care physician, and anxiety while on treatment and others (Table 4). The most troublesome non-physical concerns were almost the same as the high-frequency non-physical concerns; top of the list was ‘affects my family or partner’ followed by various anxieties and vague fears, but it is of note that the cost of medical fees ranked fifth (Table 6).

**Table 4:** Non-physical concerns noted by patients

Non-physical concerns	No. of patients (%)
Affects my family or partners	32 (65)
Feeling anxious about being unable to recover from the previous treatment	31 (63)
Affects on my work/home duties	26 (53)
Feeling sick	25 (51)
Changing the primary physician	25 (51)
Feeling anxious about the treatment	22 (45)
Feeling low	21 (43)
Feeling fearful	21 (43)
Medical costs	21 (43)
Losing the will	20 (41)
Fear of death	19 (39)
Feeling anxious about my life	18 (37)
Can return to a normal social life?	18 (37)

Among the physical symptoms, the most frequent complaints were fatigue, alopecia, constipation, loss of appetite, and paresthesia (Table 5). More than one-half of patients suffered a moderate to severe degree of fatigue, alopecia, constipation and loss of appetite. However, the most annoying problems were nausea, poor taste and paresthesia (9 patients each) followed by fatigue (8 patients), and fever and insomnia (7 patients each).

**Table 5:** Physical symptoms noted by patients

Physical symptoms	Grade of symptom^a^				
	4	3	2	1	0
Fatigue	7 (14)	13 (26)	10 (20)	6 (12)	13
Alopecia	18 (36)	6 (12)	4 (8)	4 (8)	17
Constipation	6 (12)	10 (20)	10 (20)	5 (10)	18
Loss of appetite	10 (20)	10 (20)	7 (14)	3 (6)	20
Paresthesia	10 (20)	5 (10)	5 (10)	8 (16)	21
Loss of weight	9 (18)	5 (10)	4 (8)	9 (18)	22
Shortness of breath	3 (6)	5 (10)	9 (18)	10 (20)	22
Stomatitis	5 (10)	2 (4)	5 (10)	15 (30)	22
Change in the way things taste	8 (16)	8 (16)	5 (10)	5 (10)	23
Dry skin	4 (8)	4 (8)	11 (22)	7 (14)	23
Insomnia	4 (8)	7 (14)	8 (16)	7 (14)	23
Nausea	6 (12)	2 (4)	6 (12)	10 (20)	25
Taste nothing	7 (14)	9 (18)	3 (6)	5 (10)	25
Fever	5 (10)	6 (12)	7 (14)	6 (12)	25

Values in table are presented as the number of patients with the percentage in parenthesis

^a^0, No symptoms; 1, minimal symptoms; 2, mild symptoms; 3, moderate symptoms; 4, severe symptoms

Nine patients complained of nausea as an unbearable experience. In five patients the nausea was associated with highly to moderately emetogenic anticancer drugs, while in the others the nausea was secondary to either opioid use, peritonitis carcinomatosa, tumor infiltration of the stomach or cerebral hemorrhage.

The clinical signs and symptoms that concerned the patients the most in the entire group were ‘affects my family or partner’ followed in descending order by anxiety about recovering from the previous treatment for the next therapy, anxiety about treatment, nausea, poor taste, numbness, fatigue, vague fears and so on (Table 6).

**Table 6:** Most serious concerns selected by patients from among all physical and non-physical concerns

Rank	Most serious concerns	No. of patients (%)
1	Affects my family or partner	18 (36)
2	Feeling anxious about being unable to recover from the previous therapy	13 (26)
3	Feeling anxious about treatment	10 (20)
4	Nausea	9 (18)
4	Change in the way things taste	9 (18)
4	Numbness	9 (18)
7	Fatigue	8 (16)
8	Feeling fearful	7 (14)
8	Medical costs	7 (14)
8	Fever	7 (14)
8	Insomnia	7 (14)
12	Fear of death	6 (12)
12	Anxiety to return to work	6 (12)
12	Diarrhea	6 (12)
12	Loss of appetite	6 (12)
12	Pain at infection site	6 (12)

There was no difference in symptoms between female and male patients for non-physical concerns, but for physical symptoms male patients suffered most from fever, fatigue and nausea, while females complained of poor taste, nausea, stomatitis, paresthesia, vomiting, and diarrhea (Table 7). Female patients felt that gastrointestinal problems were the most problematic side effects of chemotherapy.

**Table 7:** Difference in physical symptoms between male and female patients

Male patients		Female patients	
Symptoms	No. of patients	Symptoms	No. of patients
Fever	5	Change in the way things taste	5
Fatigue	5	Nausea	5
Nausea	4	Stomatitis	5
Paresthesia	4	Paresthesia	5
Change in the way things taste	3	Vomiting	4
Loss of appetite	3	Diarrhea	4
Insomnia	3	Pain at injection site^a^	4
		Insomnia	4

^a^Includes fear of having multiple needle sticks for chemotherapy due to poor veins

## Discussion

It is important to understand not only the significant physical symptoms but also non-physical concerns that patients feel or suffer from during systemic cancer therapy. Patients undergoing cancer chemotherapy can be made more comfortable by reducing the incidence and the severity of adverse events and by providing an appropriate dose and schedule of antineoplastic agents that will result in maximum antitumor effects with an improvement in QoL and survival.

In the early evolution of cancer chemotherapy in the 1970s and 1980s, severe CINV and emesis and nausea ranked first and second as the most troubling symptoms of cancer chemotherapy [2]. CINV in some patients was so serious that they refused to continue on their chemotherapeutic regimen despite the underlying disease being potentially curable. The situation improved with the approval of the 5HT-3 receptor antagonist as a prophylaxis for CINV in the 1990s, followed by the introduction of the NK-1 receptor antagonist at the beginning of the twenty-first century. The results of our study indicate that from the perspective of total management of cancer chemotherapy, CINV no longer ranks as a top concern among cancer patients undergoing chemotherapy.

Carelle et al. [4] reported a change in patient perceptions of the side effects of cancer chemotherapy. These authors found that ‘Affects my family or partner’ was ranked first, followed by anxieties and fears about a variety of events and non-physical concerns like ‘affects my work’ and medical costs. The results of our are in agreement with those of study agreed with those reported by Carelle et al. [4]: ‘affects my family or partner’ was the most important concern of all 94 items in the questionnaire. This result suggests that patient perceptions of the side effects or experiences during systemic chemotherapy have shifted markedly from the physical symptoms, especially from CINV, to non-physical concerns.

For physical symptoms, the number of patients with symptoms in general and number of patients with the most painful symptoms were similar. However, the significant difference in complaints about physical symptoms between male and female patients was of interest. Female patients complained of difficulty in tasting foods properly, nausea, stomatitis, and dysesthesia, while male patients suffered most from fever and fatigue. Female patients felt that gastrointestinal problems were the most problematic side effects of chemotherapy. Women in Japan are still the predominant preparer of meals for their family, but gastrointestinal symptoms would seem to preclude the preparation of meals. In particular, difficulty in appreciating sweetness and saltiness properly makes it difficult for a housewife to prepare palatable food by checking the taste during cooking. Compared with men, women experience CINV more frequently and to a more severe degree [6].

Fatigue was the most frequent symptom reported among all physical symptoms, as shown in Table 5, not only by male patients but also by female patients. It is not easy for patients to differentiate chemotherapy-induced fatigue from fatigue due to their advanced underlying disease, and this study was not designed to address that distinction [7]. Anemia is a treatable pathophysiological state that induces fatigue and weakness. It has been treated with erythropoietin in Western countries, and improvement of anemia alleviates profound fatigue [8], but erythropoietin is not approved for cancer-related anemia in Japan. Considering the incidence and severity of fatigue in cancer patients on chemotherapy, it is to be hoped that erythropoietin will be approved for clinical use in the Japanese oncology field.

As shown in Table 6, concerns about their patient’s family or partner and anxiety related to treatment were the top three complaints among our cancer patients, indicating an apparent shift from concerns regarding physical symptoms to psychosocial issues. These issues are quite subjective and difficult to measure even by appropriate instruments or tools. Therefore, unless patients express their concerns to medical staff, they may be easily overlooked or even ignored by healthcare providers. Our data and the results from a series of studies by an Australian group suggest the necessity of thorough physical symptoms and non-physical examinations before the start of chemotherapy. If a significant problem or concern is raised, it may have to be solved before treatment; otherwise, it may lead to an early interruption of the treatment and subsequent poor outcome.

Patient perceptions of the side effects of cancer chemotherapy based on the analysis of a thorough questionnaire have not previously been reported for Japanese patients. Yokoo et al. [9] carried out an Internet survey in which cancer patients’ concerns and their associations with QoL were assessed; 807 patients with all types of cancer who were older than 20 years participated in the survey, but no information on treatment was available. These authors reported that concerns about ‘self-management’ was the most common (61%) reported concern, followed by psychological symptoms (48%), medical information (46%), daily living (30%). Unfortunately concerns about family or partner were not included in this survey questionnaire. However, this Internet survey did illustrate that cancer patients in general seem to place a special emphasis on non-physical concerns rather than physical symptoms.

Patients experience a variety of side effects associated with chemotherapy. A major question to be asked by healthcare providers is whether chemotherapy is really good for patients in terms of QoL. In a prospective randomized phase three trial Dancey et al. [10] observed the superiority of second-line docetaxel over best supportive care in patients with lung cancer. The Australian–New Zealand Breast Cancer Trials Group conducted a study comparing continuous treatment with intermittent therapy in women with advanced breast cancer [11]. If disease did not progress in the patients receiving intermittent therapy, the treatment was withheld after three cycles. The same treatment was restarted only after the disease progressed, at which time three cycles were again given. This sequence was repeated until the disease no longer responded to the treatment. The survival did not differ significantly between the arms, but the QoL was worse in patients assigned to intermittent rather than continuous therapy, suggesting that QoL was improved by inducing and maintaining a tumor response despite the occurrence of side effects associated with chemotherapy.

A major limitation to our study is the small number of patients compared to previous studies, so it is difficult to draw comparisons for each gender, chemotherapy regimen, or disease, and in particular it is impossible to make statistical comparisons. However, in this study, 94 items pertinent to cancer patients undergoing chemotherapy were surveyed using a questionnaire, and important data on Japanese patients’ perception of concerns in this setting were obtained for the first time.

In conclusion, patient perceptions of adverse events associated with cancer chemotherapy have apparently shifted from physical symptoms to non-physical concerns. ‘Affects my family or partner’ is now the most important concern. A multi-disciplinary approach that includes the active involvement of medical social workers and medical staff is necessary to minimize their suffering. One interesting factor that appears not to have been noticed previously—or ignored—is the poor taste perception in female patients. This is particularly significant for Japanese women but has not been raised by women in Western studies, suggesting that the cultural background of the patient influences his/her perception of signs and symptoms associated with cancer treatment.

## Electronic supplementary material

Below is the link to the electronic supplementary material.

Supplementary material 1 (PPTX 99 kb)
