# Supplementary material for: Patient perceptions of symptoms and concerns during cancer chemotherapy: ‘affects my family’ is the most important
Source: Int J Clin Oncol. 2017 Apr 6;22(4):793–800. doi: 10.1007/s10147-017-1117-y (PMC5533818; doi:10.1007/s10147-017-1117-y)
Supplement: Supplementary file 1 — Supplementary material 1 (PPTX 99 kb) [file 10147_2017_1117_MOESM1_ESM.pptx]

## Slide 1
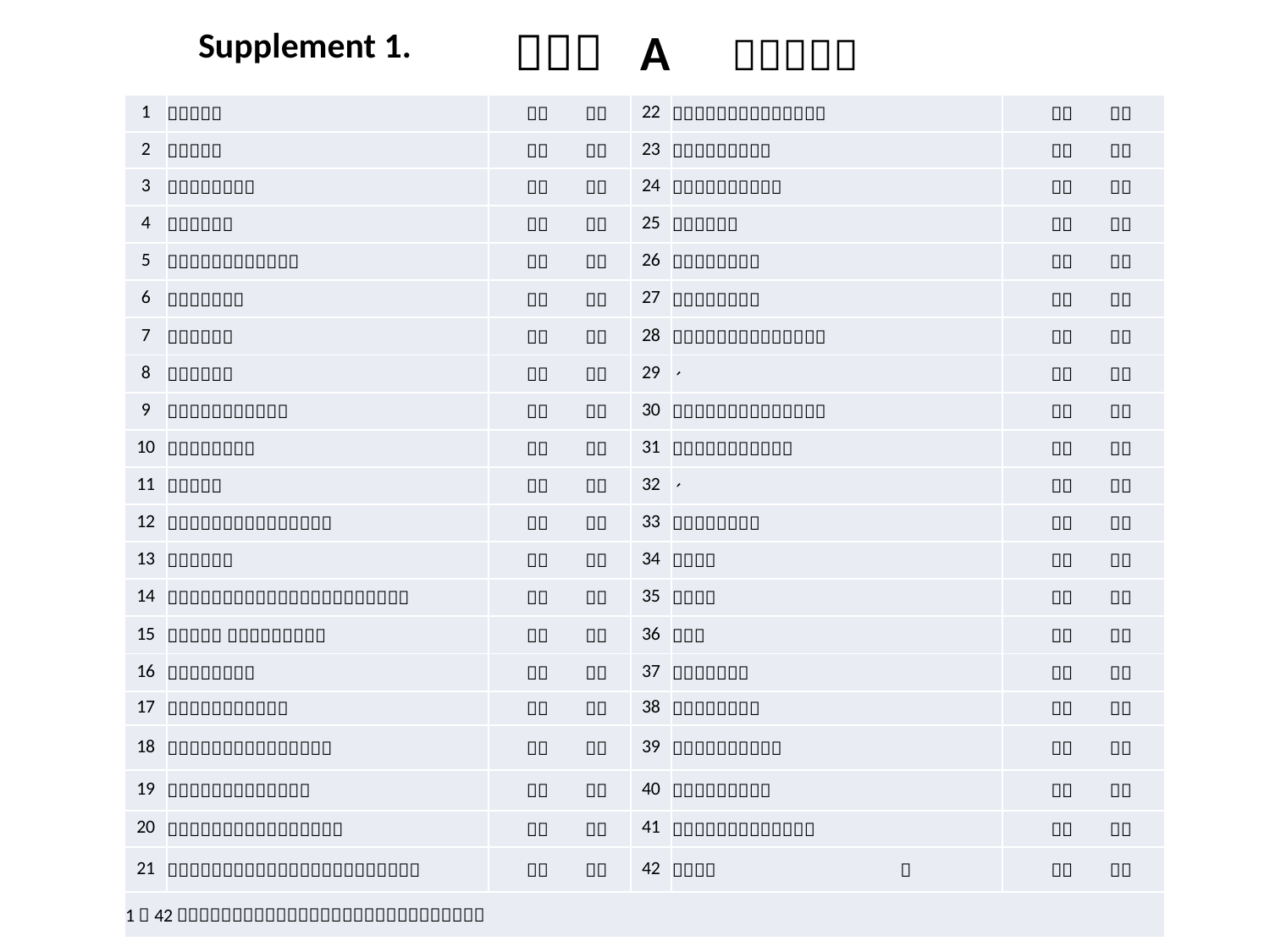

調査票 A　精神的項目
Supplement 1.
| 1 | ものわすれ | なし　　あり | 22 | ホスピスはいつ行ったらいいか | なし　　あり |
| --- | --- | --- | --- | --- | --- |
| 2 | 気分が沈む | なし　　あり | 23 | 衣類が合わなくなる | なし　　あり |
| 3 | 気分がすぐれない | なし　　あり | 24 | 仕事（家事）への影響 | なし　　あり |
| 4 | 興奮しやすい | なし　　あり | 25 | 家族への影響 | なし　　あり |
| 5 | 考え方が前向きになれない | なし　　あり | 26 | 社会復帰への不安 | なし　　あり |
| 6 | 意欲がわかない | なし　　あり | 27 | 社会活動への影響 | なし　　あり |
| 7 | メソメソする | なし　　あり | 28 | 治療にかかる時間が負担だった | なし　　あり |
| 8 | イライラする | なし　　あり | 29 | 自分以外の身内の看病、介護 | なし　　あり |
| 9 | ものごとに集中できない | なし　　あり | 30 | 生ものを食べることができない | なし　　あり |
| 10 | 漠然とした恐怖感 | なし　　あり | 31 | 結婚できるか（未婚者） | なし　　あり |
| 11 | 死への恐怖 | なし　　あり | 32 | 出産や育児、子供への影響 | なし　　あり |
| 12 | 何が起こっているか理解できない | なし　　あり | 33 | 不妊に対する不安 | なし　　あり |
| 13 | 治療への不安 | なし　　あり | 34 | 性欲減退 | なし　　あり |
| 14 | 次の治療に向けて体調が回復するかどうかの不安 | なし　　あり | 35 | 通院時間 | なし　　あり |
| 15 | 人生の不安 （漠然とした不安） | なし　　あり | 36 | 医療費 | なし　　あり |
| 16 | 相談相手がいない | なし　　あり | 37 | 医療者との関係 | なし　　あり |
| 17 | 支えてくれる人がいない | なし　　あり | 38 | 他の患者との関係 | なし　　あり |
| 18 | 人に病気のことを知られたくない | なし　　あり | 39 | 医療機関とのトラブル | なし　　あり |
| 19 | 主治医が変わることへの不満 | なし　　あり | 40 | 医療者への連絡方法 | なし　　あり |
| 20 | インターネットなどの情報の信用性 | なし　　あり | 41 | 医療者に連絡してもいいのか | なし　　あり |
| 21 | セカンドオピニオンを受けたいが主治医に気を遣う | なし　　あり | 42 | その他（　　　　　　　　　　） | なし　　あり |
| 1～42番の中で辛かった悩んだ項目を上位３つ番号を選んでください | | | | | |

## Slide 2
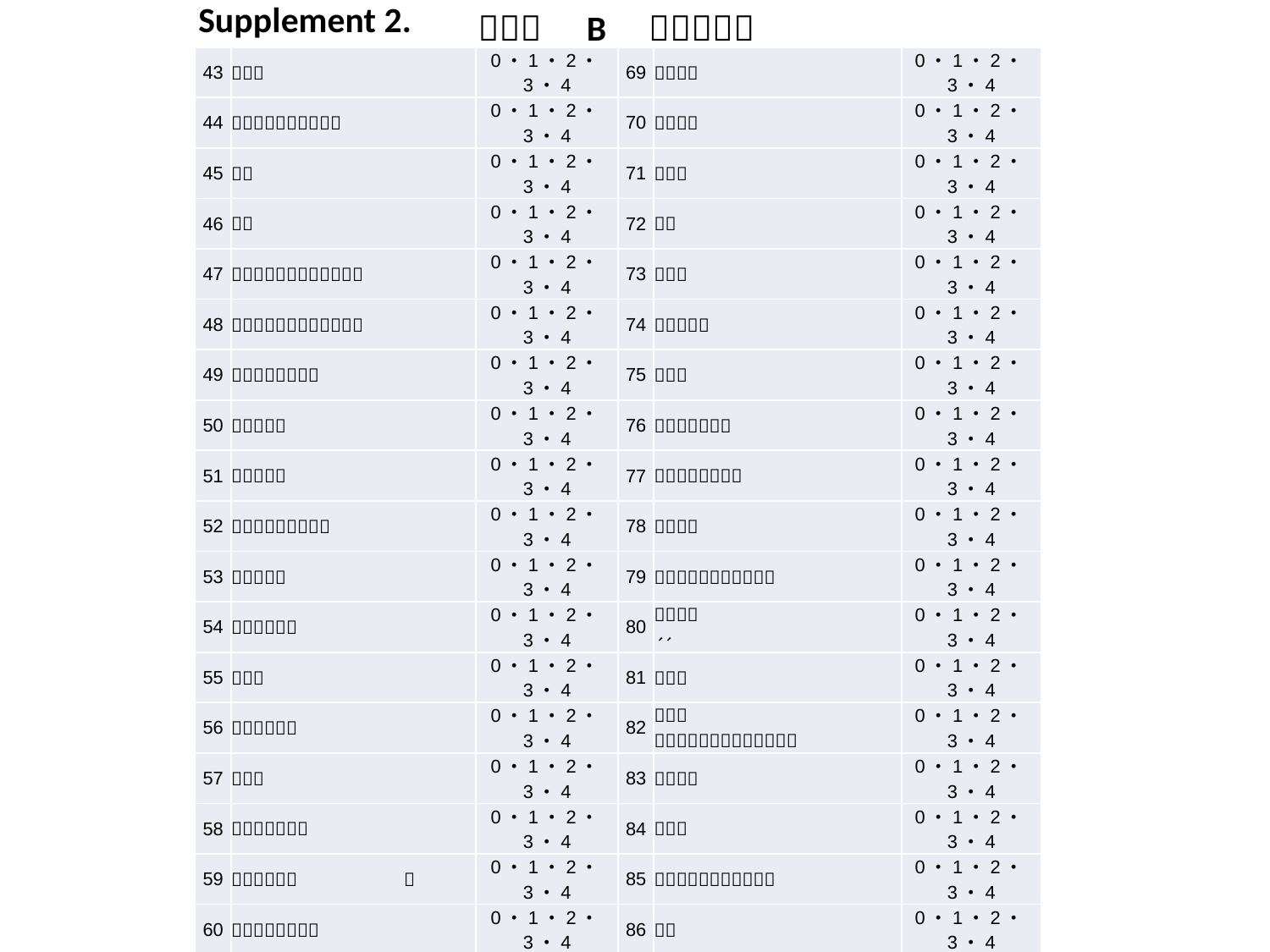

Supplement 2.
調査票　B　身体的項目
| 43 | 吐き気 | 0・1・2・3・4 | 69 | 排尿時痛 | 0・1・2・3・4 |
| --- | --- | --- | --- | --- | --- |
| 44 | 嘔吐（実際に吐いた） | 0・1・2・3・4 | 70 | 排尿困難 | 0・1・2・3・4 |
| 45 | 便秘 | 0・1・2・3・4 | 71 | 鼻出血 | 0・1・2・3・4 |
| 46 | 下痢 | 0・1・2・3・4 | 72 | 頭痛 | 0・1・2・3・4 |
| 47 | 味覚（味）がいつもと違う | 0・1・2・3・4 | 73 | めまい | 0・1・2・3・4 |
| 48 | 食べ物の味がしなくなった | 0・1・2・3・4 | 74 | たちくらみ | 0・1・2・3・4 |
| 49 | 食欲が無くなった | 0・1・2・3・4 | 75 | 耳鳴り | 0・1・2・3・4 |
| 50 | のどの痛み | 0・1・2・3・4 | 76 | 耳の聴こえ難さ | 0・1・2・3・4 |
| 51 | お腹の痛み | 0・1・2・3・4 | 77 | あざができやすい | 0・1・2・3・4 |
| 52 | お腹がはってきつい | 0・1・2・3・4 | 78 | じんま疹 | 0・1・2・3・4 |
| 53 | のどの渇き | 0・1・2・3・4 | 79 | 肌の色の変化（黒ずみ） | 0・1・2・3・4 |
| 54 | においの変化 | 0・1・2・3・4 | 80 | 爪の変化 （色、痛み、割れたなど） | 0・1・2・3・4 |
| 55 | 口内炎 | 0・1・2・3・4 | 81 | ほてり | 0・1・2・3・4 |
| 56 | 食欲が増した | 0・1・2・3・4 | 82 | にきび （皮疹：湿疹のようなもの） | 0・1・2・3・4 |
| 57 | 口角炎 | 0・1・2・3・4 | 83 | 肌の乾燥 | 0・1・2・3・4 |
| 58 | 飲み込みにくい | 0・1・2・3・4 | 84 | かゆみ | 0・1・2・3・4 |
| 59 | しびれ（部位　　　　　 ） | 0・1・2・3・4 | 85 | 日光アレルギー様皮膚炎 | 0・1・2・3・4 |
| 60 | 手先・足先の痛み | 0・1・2・3・4 | 86 | 発熱 | 0・1・2・3・4 |
| 61 | 感覚の鈍さ | 0・1・2・3・4 | 87 | 倦怠感（体がきつい） | 0・1・2・3・4 |
| 62 | 知覚過敏（部位　　　　　 ） | 0・1・2・3・4 | 88 | 脱毛 | 0・1・2・3・4 |
| 63 | 注射部位の痛み | 0・1・2・3・4 | 89 | 体重減少 | 0・1・2・3・4 |
| 64 | 血管が出ない不安/恐怖 | 0・1・2・3・4 | 90 | 体重増加 | 0・1・2・3・4 |
| 65 | 関節痛 | 0・1・2・3・4 | 91 | むくみ | 0・1・2・3・4 |
| 66 | 筋肉痛 | 0・1・2・3・4 | 92 | 動悸 | 0・1・2・3・4 |
| 67 | 尿の色 | 0・1・2・3・4 | 93 | 息切れ | 0・1・2・3・4 |
| 68 | 尿量の変化 | 0・1・2・3・4 | 94 | 不眠 | 0・1・2・3・4 |
| 43～94番の中で辛かった悩んだ項目を上位３つ番号を選んでください | | | | | |
